# Comparative impact of imipenem and meropenem on intestinal carriage and relative fecal abundance of multidrug-resistant bacteria, with detection of residual antibiotics in rectal swabs

**DOI:** 10.1128/spectrum.01235-26

**Published:** 2026-09-17

**Authors:** Matthieu Holub, Assaf Mizrahi, Olivier Miot, Gauthier Péan de Ponfilly, Pauline Huriez, Laurène Cachera, Thibaut Genty, Jean-Ralph Zahar, Nathalie Leveque, Alban Le Monnier, Benoit Pilmis

**Affiliations:** 1 Equipe mobile de Microbiologie clinique, Hôpitaux Saint Joseph & Marie Lannelongue 55662 , Paris, France; 2 Plateforme des dosages des anti-infectieux, Hôpitaux Saint Joseph & Marie Lannelongue 55662 , Paris, France; 3 Service de Microbiologie Clinique, Hôpitaux Saint Joseph et Marie Lannelongue 55662 , Paris, France; 4 Micalis Institute UMR 1319, Université Paris-Saclay, INRAE, AgroParisTech, Jouy-En-Josas, France; 5 Service de Réanimation, Hôpitaux Saint-Joseph & Marie-Lannelongue, Le Plessis-Robinson, France; 6 INSERM, IAME UMR 1137, University of Paris, Paris, France; 7 Microbiology, Infection Control Unit, GH Paris Seine Saint-Denis, APHP, Bobigny, France; Quest Diagnostics Nichols Institute, Chantilly, Virginia, USA

**Keywords:** carbapenem, multidrug-resistant bacteria, relative fecal abundance

## Abstract

Carbapenems are widely used in critically ill patients, yet their impact on intestinal carriage and relative fecal abundance (RFA) of *Enterobacterales*, *Enterococcus* spp., *Pseudomonas aeruginosa*, and *Clostridioides difficile* remains poorly characterized. We compared the effects of imipenem and meropenem on these targeted bacterial populations and on carriage of resistant organisms. Eighty-three hospitalized patients receiving imipenem (*n* = 52) or meropenem (*n* = 31) were included. Rectal samples were collected before treatment (T0), at 48–72 h (T1), and at the end of therapy (T2). Culture-based methods assessed changes in cultivable species, RFA, and carriage of multidrug-resistant organisms. Fecal carbapenem residues and degradation products were quantified by ultra-high performance liquid chromatography–high-resolution mass spectrometry. Longitudinal changes were compared between treatment groups and according to fecal antibiotic detection. At T1, RFA of third-generation cephalosporin-resistant *Enterobacterales* (3GCR-E) was higher with meropenem than imipenem (95% vs 32%, *P* = 0.007), with no difference at T2. Between T0 and T1, the number of cultivable *Enterobacterales* species decreased more frequently in the meropenem group (57.1% vs 21.9%, *P* = 0.018). Antibiotic was detected in rectal samples of 59.1% of meropenem—vs 25.8% of imipenem-treated patients; the most marked changes occurred in meropenem-treated patients with detectable residues. 3GCR-E carriage persisted under both treatments at T2 (imipenem 28.6%, meropenem 58.3%, *P* = 0.092). Imipenem was associated with lower intestinal exposure and more limited changes in targeted cultivable intestinal bacterial populations than meropenem. Pharmacokinetic differences may drive differential intestinal microbiological effects of carbapenems, supporting stewardship strategies that account for intestinal consequences.

**IMPORTANCE:**

Carbapenems are essential for the treatment of severe infections caused by multidrug-resistant bacteria, yet their intestinal microbiological consequences remain insufficiently characterized in clinical practice. This study provides direct clinical evidence that imipenem and meropenem, despite similar antibacterial spectra, differ in their intestinal exposure and in their association with changes in targeted cultivable intestinal bacterial populations. By combining culture-based microbiological analyses with direct quantification of fecal carbapenem residues, we demonstrate that meropenem is more frequently detected in the gut and is associated with greater reductions in cultivable Enterobacterales diversity, whereas imipenem is associated with more limited microbiological changes. These findings underscore the relevance of pharmacokinetic determinants and support antimicrobial stewardship strategies that integrate intestinal microbiological consequences alongside clinical efficacy, particularly in high-risk hospitalized patients. These differences were most marked during the first days of therapy, identifying the early treatment interval as the period of greatest perturbation of the intestinal reservoir.

**CLINICAL TRIALS:**

This study is registered with ClinicalTrials.gov as NCT05516433.

## INTRODUCTION

Antibiotic resistance represents a major global health challenge that threatens the effectiveness of anti-infective therapies worldwide. In 2019, antimicrobial resistance (AMR) was estimated to be associated with 4.95 million deaths, including 1.27 million directly attributable deaths (1). Among resistant pathogens, third-generation cephalosporin-resistant Enterobacterales (3GCR-E) and carbapenem-resistant Enterobacterales (CRE) are of particular concern because they are associated with increased morbidity, mortality, and additional healthcare costs (2, 3). Intestinal carriage of multidrug-resistant (MDR) organisms is an important epidemiological reservoir and contributes to both subsequent infections and cross-transmission in hospitalized patients (4, 5).

The intestinal microbiota plays a central role in colonization resistance against opportunistic pathogens (6, 7). Antibiotic exposure can impair this resistance and promote intestinal carriage or overgrowth of MDR organisms (8, 9). However, the ecological effects of antibiotics depend not only on their antibacterial spectrum (10) but also on pharmacokinetic and pharmacodynamic determinants, particularly intestinal exposure, biliary excretion, and residual fecal antibiotic concentrations (11, 12). Thus, antibiotics with apparently narrow spectra may exert marked intestinal ecological effects when fecal exposure is high (13), whereas broader-spectrum agents may have a more limited intestinal impact when fecal exposure is low (14, 15).

Carbapenems are cornerstone agents for the treatment of severe infections caused by extended-spectrum beta-lactamase (ESBL)-producing Enterobacterales. Among them, imipenem and meropenem are widely used in clinical practice and display overlapping antibacterial activity against Enterobacterales and *Pseudomonas aeruginosa* (16). However, their pharmacological profiles differ substantially. Imipenem is characterized by minimal biliary elimination (approximately 0.3%) and limited fecal excretion (around 2%), whereas meropenem demonstrates higher biliary diffusion (up to 25%) and greater fecal excretion (reaching up to 5%) (14, 15, 17, 18). In addition, imipenem has stronger intrinsic activity against *Enterococcus faecalis*, while meropenem generally exhibits lower minimum inhibitory concentrations (MICs) against Gram-negative pathogens (16, 19–21). These pharmacological and microbiological differences may result in distinct effects on intestinal bacterial populations.

Experimental and clinical data have suggested that imipenem may have a limited intestinal ecological impact (17, 22–25), whereas meropenem exposure has been associated with reductions in anaerobic populations and overgrowth of *Enterococcus* spp. or other resistant pathobionts (26–28). Nevertheless, the available evidence remains heterogeneous and is often derived from small cohorts, studies in healthy volunteers, or culture-based investigations. In addition, direct comparative data between imipenem and meropenem in hospitalized patients remain scarce (11, 29).

In this context, comparative studies are needed to better characterize the differential effects of carbapenems on intestinal carriage and targeted cultivable intestinal bacterial populations and their potential role in the selection of MDR organisms. The MERIMI study was designed to address this gap by evaluating, in hospitalized patients, the comparative impact of imipenem and meropenem on (i) intestinal carriage of MDR organisms, (ii) changes in cultivable intestinal bacterial populations, assessed by culture-based species counts, (iii) the relative fecal abundance (RFA) of resistant Enterobacterales, and (iv) the detection of residual antibiotics in rectal swabs.

## MATERIALS AND METHODS

### Study design

The MERIMI study was a prospective, multicenter observational study conducted between January 2023 and June 2025 at Paris Saint-Joseph Hospital and Marie Lannelongue Hospital, France. Eligible patients were adults (≥18 years) who were hospitalized and received imipenem or meropenem as either empirical or targeted therapy for suspected or documented infection. Exclusion criteria were (i) known hypersensitivity to carbapenems; (ii) exposure to any carbapenem within the previous four months; (iii) pregnancy or breastfeeding; and (iv) legal guardianship, curatorship, or deprivation of liberty. Clinical data were extracted from electronic medical records from hospital admission to discharge and entered into an anonymized case report form (CRF).

### Sampling procedure

Rectal swabs (FecalSwab, Copan Diagnostics, Murrieta, CA, USA) were collected at three predefined time points: at inclusion, immediately before carbapenem infusion (T0); 48–72 h after treatment initiation (T1); and at carbapenem discontinuation or switch to oral therapy (T2) (Fig. S1). Each FecalSwab device contained 2 mL of modified Cary–Blair medium. Samples were stored at 4°C and processed within 24–48 h whenever feasible; otherwise, they were stored at −80°C until analysis. After homogenization by centrifugation (2–3 min), the transport medium was aliquoted as follows: 100 µL for carriage and cultivable bacterial analyses, and 200 µL for residual antibiotic testing.

### Microbiological analysis

An aliquot of 100 µL of transport medium was enriched in 9.9 mL of brain–heart infusion (BHI) broth supplemented with 10% glycerol and incubated overnight at 35°C. Thereafter, 10 µL of the enriched suspension was plated with a calibrated loop onto six agar media, using serial dilutions from 10⁻¹ to 10⁻⁵: Five of the six media were chromogenic. chromID CPS Elite (bioMérieux, Marcy-l’Etoile, France) is a chromogenic, non-inhibitory medium containing no antimicrobial supplement, used to enumerate total aerobic cultivable bacteria and enterococci and to provide presumptive identification. Drigalski agar (Bio-Rad Laboratories, Hercules, CA, USA) is a non-chromogenic, lactose-based medium selective for Gram-negative bacilli, used to enumerate total cultivable *Enterobacterales*. chromID ESBL agar (bioMérieux), chromID CARBA/OXA agar (bioMérieux), and chromID VRE agar (bioMérieux) are chromogenic media rendered selective by, respectively, cefpodoxime, carbapenem, and vancomycin supplementation and were used to screen for third-generation cephalosporin-resistant *Enterobacterales*, carbapenemase-producing *Enterobacterales*, and vancomycin-resistant enterococci. chromID *C. difficile* (CDIF) agar (bioMérieux) is a taurocholate-supplemented chromogenic selective medium for the detection and identification of *Clostridioides difficile*. All plates were incubated overnight at 35°C under aerobic conditions, except chromID *C. difficile*, which was incubated anaerobically. No non-selective, non-chromogenic medium (e.g., Columbia blood agar) and no anaerobic culture other than for *C. difficile* were performed. Throughout this manuscript, the targeted bacterial populations refer to the organism groups covered by this culture panel: *Enterobacterales*, *Enterococcus* spp., *Pseudomonas aeruginosa*, and *Clostridioides difficile*. No other bacterial population was sought or quantified.

All distinct colony morphotypes recovered on each medium were subcultured and identified by matrix-assisted laser desorption/ionization time-of-flight mass spectrometry (MALDI-TOF MS; Bruker Daltonics, Bremen, Germany) irrespective of chromogenic color. Identification was, therefore, not restricted to organisms included in the chromogenic identification schemes of the media used. Identification was, therefore, not restricted to organisms included in the chromogenic identification schemes of the media used.

Antimicrobial susceptibility testing was performed by disk diffusion on Mueller–Hinton agar (Bio-Rad) according to the recommendations of the Comité de l’Antibiogramme de la Société Française de Microbiologie (CA-SFM v1.0, 2022). Inhibition zone diameters were interpreted according to CA-SFM/EUCAST clinical breakpoints and measured using the Sirweb/Sirscan automated system (i2a, Montpellier, France).

*Enterobacterales* isolates were screened for third-generation cephalosporin resistance using CA-SFM/EUCAST screening cut-offs (ceftazidime inhibition zone diameter ≤22 mm and/or cefotaxime ≤20 mm and/or cefotaxime MIC ≥2 mg/L) (30). Isolates meeting these criteria were further characterized phenotypically as follows.

ESBL production was assessed by the double-disk synergy test (31), with amoxicillin–clavulanic acid (20/10 µg) disks placed 30 mm center-to-center from cefotaxime (5 µg), ceftazidime (10 µg), and cefepime (30 µg) disks; a characteristic synergy image was considered positive. In species with inducible chromosomal AmpC (*Enterobacter cloacae* complex, *Klebsiella aerogenes*, *Citrobacter freundii* complex, *Serratia* spp., *Morganella morganii*, *Providencia* spp., *Hafnia alvei*) and in cefoxitin-resistant isolates, in which the synergy image may be masked, ESBL production was assessed by the combined-disk method comparing cefepime (30 µg) alone with cefepime plus clavulanic acid (30/10 µg); an increase in inhibition zone diameter of ≥5 mm was considered positive.

AmpC overproduction was defined phenotypically by restoration of the inhibition zone around cefotaxime or ceftazidime on Mueller–Hinton agar supplemented with cloxacillin (Bio-Rad). β-Lactamase genes were not characterized molecularly; all classifications reported here are phenotypic.

Carbapenemase detection was performed according to the CA-SFM/EUCAST v1.0 (2022) algorithm and confirmed using the OKNVI immunochromatographic test (Coris BioConcept, Gembloux, Belgium). This assay detects the five most prevalent carbapenemases (OXA-48-like, KPC, NDM, VIM, and IMP) and does not exclude the presence of a less common enzyme; no molecular characterization was performed. *Clostridioides difficile* toxin production was assessed using an enzyme immunoassay (C. Diff Quik Chek Complete, TechLab, Blacksburg, VA, USA).

Colonies presumptively identified as enterococci on chromID CPS Elite agar were identified to species level by MALDI-TOF MS. Because *Enterococcus faecalis* is almost uniformly susceptible to aminopenicillins, whereas *Enterococcus faecium* is frequently resistant, aminopenicillin susceptibility testing was restricted to *E. faecium* isolates. Susceptibility was determined by disk diffusion on Mueller–Hinton agar using a 2 µg ampicillin disk, with a 0.5 McFarland inoculum and overnight incubation at 35°C, in accordance with CA-SFM/EUCAST recommendations for *Enterococcus* spp.; isolates are reported here as aminopenicillin-susceptible or aminopenicillin-resistant. Inhibition zone diameters were interpreted using CA-SFM (2022) breakpoints. *Enterococcus faecalis* ATCC 29212 was used as the quality control strain. Vancomycin-resistant enterococci were screened separately on chromID VRE agar.

The number of cultivable species was determined on the six media used, and culture abundance was expressed as colony-forming units (CFU)/mL. Total cultivable *Enterobacterales* were enumerated on Drigalski agar; enterococci and aerobic cultivable bacteria were enumerated on chromID CPS Elite. Because this medium has not been validated for fecal matrices, counts obtained on it are reported throughout as aerobic cultivable bacteria recovered on chromID CPS Elite, and not as total aerobic bacteria.

A loss of cultivable species between two time points was defined as the disappearance of at least one identified species, whereas a decrease in culture abundance was defined as a reduction of at least 2 log₁₀ CFU/mL.

The relative fecal abundance (RFA) of 3GCR-E was calculated using two denominators: total cultivable *Enterobacterales* recovered on Drigalski agar, which corresponds to the definition used in previous studies (11, 32, 33) and was considered the primary measure; aerobic cultivable bacteria recovered on chromID CPS Elite, considered a secondary, exploratory measure.

Acquisition of a resistant micro-organism was defined as its detection at T1 or T2 in a patient from whom it had not been recovered at T0.

### Pharmacological analysis

Residual antibiotic analyses were performed on 200 µL of transport medium using ultra-high-performance liquid chromatography coupled to high-resolution mass spectrometry (UHPLC-HRMS; Ultimate 3000 coupled to an Orbitrap Exactive Plus, Thermo Scientific). Imipenem (*m*/*z* 300.1012) and meropenem (*m*/*z* 384.1580) were quantified using stable isotope-labeled internal standards (D₄-imipenem and D₆-meropenem, respectively). Calibration standards and quality control (QC) samples were prepared in advance, included in each analytical run, and stored at −80°C until use. After addition of internal standards and two successive centrifugation steps, the supernatants were diluted in LC–MS-grade water and injected in volumes ranging from 10 to 20 µL. Chromatographic separation was achieved using a TurboFlow Cyclone-MCX2 column for online sample cleanup, followed by a Hypersil GOLD Aq column, with gradient elution using acetonitrile. In addition, the hydrolysis product of each carbapenem was monitored as a complementary marker of drug presence in the sample. For imipenem, this corresponded to the hydrolysis derivative at *m*/*z* 318.1123 (C₁₃H₂₀N₃O₅S). As no analytical standard was available for these compounds, they were reported as detected or not detected rather than quantified. The analytical method was developed and validated as described elsewhere (34).

### Statistical analysis

For descriptive analyses, all available samples were included provided that at least one sample had been collected. Longitudinal analyses included only patients with at least one evaluable baseline sample (T0) and at least one subsequent follow-up sample. Patients contributing an evaluable T0 sample and at least one evaluable follow-up sample (T1 or T2) are referred to throughout as patients with paired samples. Continuous variables were expressed as medians with interquartile ranges (IQRs) and compared using Student’s *t* test or the Mann–Whitney *U* test, as appropriate. Categorical variables were expressed as counts and percentages (*n*, %) and compared using the *χ*² test or Fisher’s exact test. Paired comparisons across time points were performed using the Wilcoxon signed-rank test. A *P* value < 0.05 was considered statistically significant. Statistical analyses were conducted using R software version 4.4.2 (R Foundation for Statistical Computing).

### Quality control and reproducibility

Microbiological procedures were standardized across both study sites. Internal controls and calibration curves were systematically included in all pharmacological assays to ensure analytical accuracy and reproducibility.

## RESULTS

### Study population and baseline characteristics

**Fig 1:**
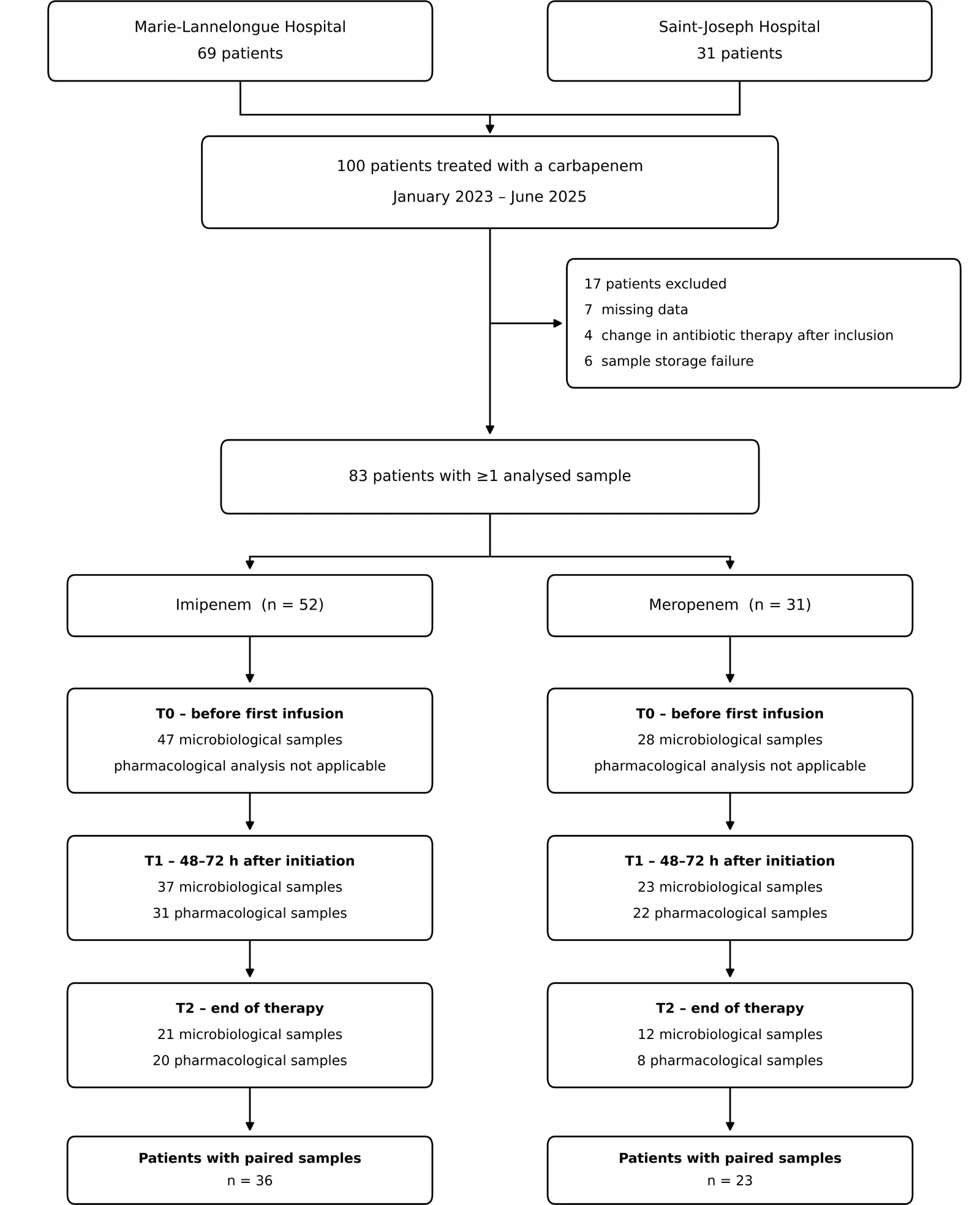
Study flowchart.

Among the 83 included patients, 52 (63%) received imipenem and 31 (37%) received meropenem (Fig. 1). Baseline characteristics were broadly similar between the two treatment groups. However, patients treated with imipenem were more frequently admitted to the intensive care unit than those treated with meropenem (88.5% vs 67.7%, *P* = 0.021). In addition, the median defined daily dose was higher in the meropenem group than in the imipenem group (3 g/day [IQR 3–4]) vs (2 g/day [IQR 2–2], *P* < 0.001) (Table 1). Concomitant antibiotic therapy was administered to 29 of 52 patients (55.8%) in the imipenem group and 14 of 31 (45.2%) in the meropenem group. Co-administered agents were predominantly aminoglycosides. Agents active against Gram-positive organisms were also administered: linezolid in nine patients, daptomycin in five, and intravenous vancomycin in five. No patient received a fluoroquinolone, metronidazole, or an oral glycopeptide during the study period.

**TABLE 1:** Characteristics of patients, infections, and treatments

Characteristic	Total *n* = 83	Imipenem *n* = 52	Meropenem *n* = 31	*P*-value
General characteristics				
Age, years, median [IQR]	66 [55–74]	65 [55–75]	67 [54–73]	0.205
Female sex, *n* (%)	21 (25.3)	16 (30.8)	5 (16.1)	0.138
Weight, kg, median [IQR]	77 [69–86]	77 [70–86]	77 [65–86]	0.636
ICU admission, *n* (%)	67 (80.7)	46 (88.5)	21 (67.7)	0.021
Length of stay, days, median [IQR]	21 [8–44]	24 [8–45]	15 [7–44]	0.298
Source of infection				
Respiratory tract infection, *n* (%)	46 (55.4)	32 (61.5)	14 (45.2)	0.175
Urinary tract infection, *n* (%)	12 (14.5)	6 (11.5)	6 (19.4)	0.350
Intra-adbominal infection, *n* (%)	5 (6.0)	1 (1.9)	4 (12.9)	0.062
Skin/soft tissue infection, *n* (%)	8 (9.6)	3 (5.8)	5 (16.1)	0.142
Catheter-related infection, *n* (%)	8 (9.6)	7 (13.5)	1 (3.2)	0.248
Mediastinitis, *n* (%)	4 (4.8)	3 (5.8)	1 (3.2)	1.000
Antibiotic therapy				
Prior antibiotic use (<4 months), *n* (%)	80 (96.4)	49 (94.2)	31 (100)	0.173
Time to carbapenem, days, median [IQR]	8 [2–14]	7 [2–13]	9 [2–16]	0.161
Combination therapy, *n* (%)	43 (51.8)	29 (55.8)	14 (45.2)	0.479
Carbapenem as empirical therapy, *n* (%)	39 (46.9)	28 (53.8)	11 (35.9)	0.163
Carbapenem as definitive therapy, *n* (%)	62 (74.4)	35 (67.3)	27 (87.1)	0.081
Daily dose, g, median [IQR]	2 [2–3]	2 [2–2]	3 [3–4]	<0.001
Duration, days, median [IQR]	7 [4–7]	7 [4–7]	5 [3–7]	0.992

### Baseline carriage and cultivable bacterial measures

At baseline (T0), carriage rates of third-generation cephalosporin-resistant Enterobacterales (3GCR-E), carbapenem-resistant Enterobacterales (CRE), *Pseudomonas aeruginosa*, and aminopenicillin-resistant *Enterococcus faecium* were similar between the two treatment groups. Baseline numbers of cultivable species and culture abundance did not differ significantly between groups (Table 2; Tables S1 and S2). At T1, aminopenicillin-resistant *E. faecium* was recovered from 9 of 16 patients in the imipenem group in whom *E. faecium* was detected, compared with 14 of 14 in the meropenem group (*P* = 0.007) (Table 2). No vancomycin-resistant enterococci were detected at any time point. The median duration of carbapenem therapy before T2 sample collection was seven days [IQR 6–8] in the imipenem group and seven days [IQR 5–13] in the meropenem group.

**TABLE 2:** Microbiological characteristics at different time points^*a*^

Characteristics	Imipenem	Meropenem	*P*-value
**T0—Baseline (*****n*** **= 47 vs 28)**			
Carriage			
3GCR-E, *n* (%)	26 (55.3)	17 (60.7)	0.648
AmpC producing *Enterobacterales*	5 (19.2)	3 (17.3)	
ESBL producing *Enterobacterales*	21 (80.8)	14 (82.3)	
Carbapenem-resistant *Enterobacterales*, *n* (%)	1 (2.1)	1 (3.6)	1
*Clostridioides difficile*	0 (0)	0 (0)	NA
Vancomycin*-resistant enterococci*, *n* (%)	0 (0)	0 (0)	NA
Aminopenicillin-resistant *Enterococcus faecium*, *n* (%)	16 (66.7)	7 (87.5)	0.386
*Pseudomonas aeruginosa*	3 (6.4)	4 (14.3)	0.413
Including carbapenem resistant	1 (66.7)	1 (25.0)	
Cultivable species			
Total, no. of species, median [IQR]	3 [2.8–3]	3 [2.8–3]	0.931
E*nterobacterales,* no. of species, median [IQR]	1 [1–2]	1.5 [1–2]	0.353
Culture abundance			
Total abundance (log10), median [IQR]	8.7 [8.3–8.9]	8.7 [8.1–9.0]	0.565
Abundance in *Enterobacterales* (log10), median [IQR]	7.6 [6.8–8.4]	8 [6.8–8.4]	0.977
Abundance in *enterococci* (log10), median [IQR]	8.5 [7.3–8.7]	8.4 [7.3–8.8]	0.841
RFA in 3GCR-E (%)	17.5 [2.5–50.8]	24.5 [6.8–47.8]	0.488
**T1—48–72 h (*****n*** **= 37 vs 23)**			
Carriage			
3GCR-E, *n* (%)	18 (48.6)	11 (47.8)	0.951
Including AmpC	2 (11.1)	3 (27.2)	
Including ESBL	16 (88.8)	8 (72.2)	
Carbapenem-resistant Enterobacterales, *n* (%)	2 (5.4)	1 (4.3)	1
*Clostridioides difficile, n* (%)	0 (0)	1 (4.3)	0.383
Vancomycin-resistant enterococci, *n* (%)	0 (0)	0 (0)	NA
Aminopenicillin-resistant *Enterococcus faecium*, *n* (%)	9 (56.2)	14 (100)	0.007
*P. aeruginosa, n* (%)	3 (8.1)	1 (4.3)	1
Including carbapenem resistant	2 (66.7)	0 (0)	
Cultivable species			
Total, no. of species, median [IQR]	2 [1–3]	2 [2–3]	0.937
*Enterobacterales,* no. of species, median [IQR]	1 [0–1]	1 [0–1]	0.484
Culture abundance			0.994
Total abundance (log10), median [IQR]	8.7 [8.5–8.8]	8.7 [7.4–8.9]	
Abundance in *Enterobacterales* (log10), median [IQR]	8.2 [6.8–8.6]	7.7 [6.9–8.1]	0.404
Abundance in *enterococci* (log10), median [IQR]	8.2 [7.6–8.7]	8.2 [7.5–8.6]	0.804
RFA in 3GCR-E (%), median [IQR]	32 [8.5–59]	95 [70.5–100]	0.008
**T2—End of therapy (*****n*** **= 21 vs 12)**			
Carriage			
3GCR-E, *n* (%)	6 (28.6)	7 (58.3)	0.092
Including AmpC	6 (100)	5 (71.4)	
Including ESBL	0 (0)	2 (28.6)	
Carbapenem-resistant Enterobacterales, *n* (%)	1 (4.8)	0 (0)	1
*Clostridioides difficile*, *n* (%)	0 (0)	0 (0)	NA
Vancomycin-resistant enterococci, *n* (%)	0 (0)	0 (0)	NA
Aminopenicillin-resistant *Enterococcus faecium*, *n* (%)	8 (80.0)	6 (85.7)	1
*Pseudomonas aeruginosa*	2 (9.5)	1 (8.3)	1
Including carbapenem resistant	2 (100)	0 (0)	
Cultivable species			
Total, no. of species, median [IQR]	2 [2–3]	2 [2–2]	0.241
*Enterobacterales,* no. of species, median [IQR]	1 [1–2]	1 [0–1.2]	0.382
Culture abundance			
Total abundance (log10), median [IQR]	8.9 [8.3–9.1]	9 [8.7–9.2]	0.489
Abundance in *enterobacterales* (log10), median [IQR]	8 [6.8–8.7]	7.9 [6.7–9.2]	0.787
Abundance in *enterococci* (log10), median [IQR]	8.7 [7.8–8.8]	8.7 [8.5–8.9]	0.565
RFA in 3GCR-E (%), median [IQR]	11.5 [5–36]	40 [10.5–82]	0.144

^*a*^ NA, not assessed.

### Cross-sectional abundance of 3GCR-Enterobacterales

**Fig 2:**
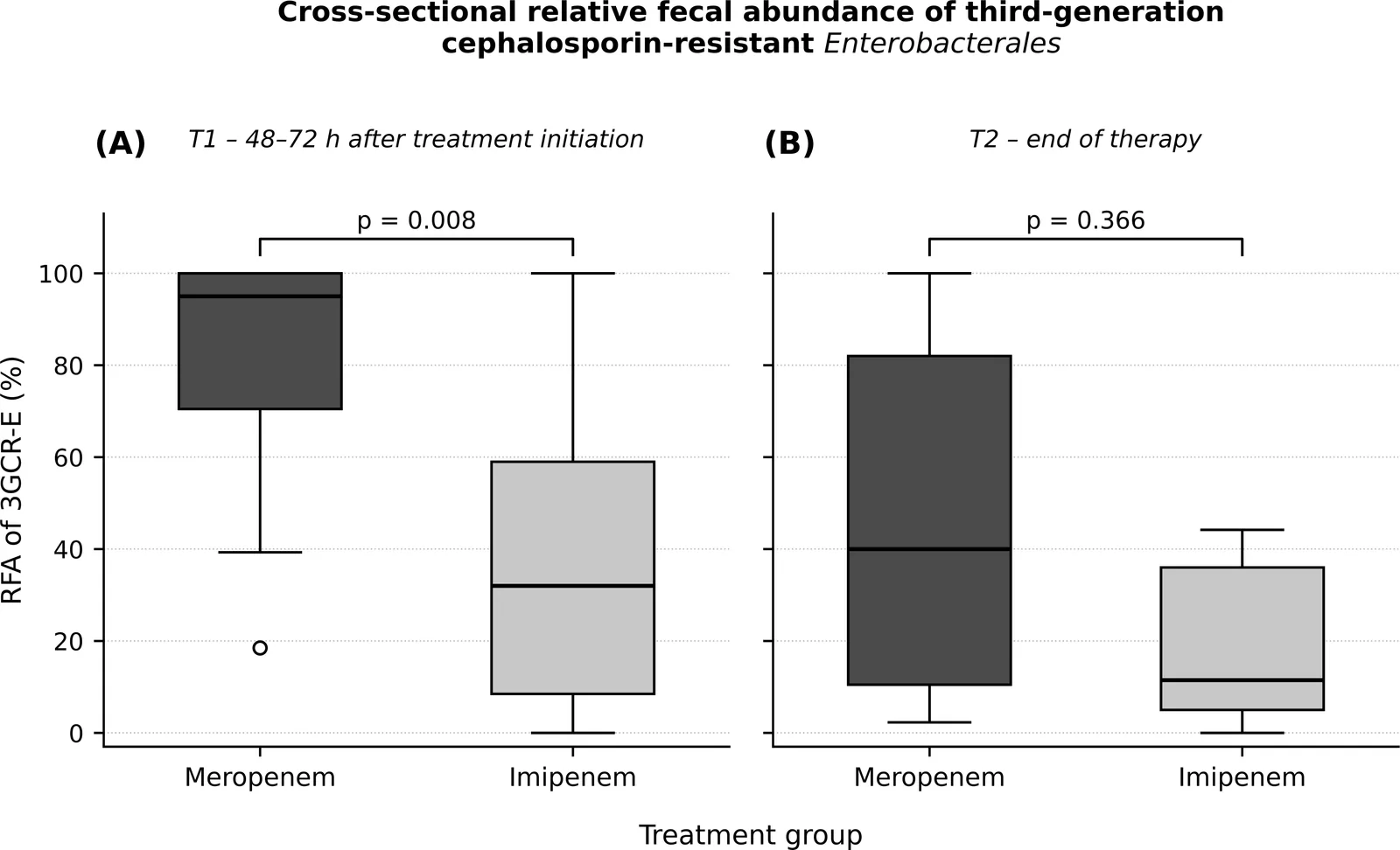
Relative fecal abundance of 3CGR-E, by treatment group. (**A**) At initiation therapy. (**B**) T2-end of therapy.

At T1 (48–72 h), the relative fecal abundance (RFA) of 3GCR-E was significantly higher in patients treated with meropenem than in those treated with imipenem (95% [IQR 71–100] vs 32% [IQR 9–59]; *P* = 0.008) (Fig. 2A). By T2 (end of therapy; median duration, seven days), RFA values were no longer significantly different between groups (40% [IQR 11–83] versus 12% [IQR 6–37]; *P* = 0.366) (Fig. 2B).

### Longitudinal changes in cultivable bacterial measures

**Fig 3:**
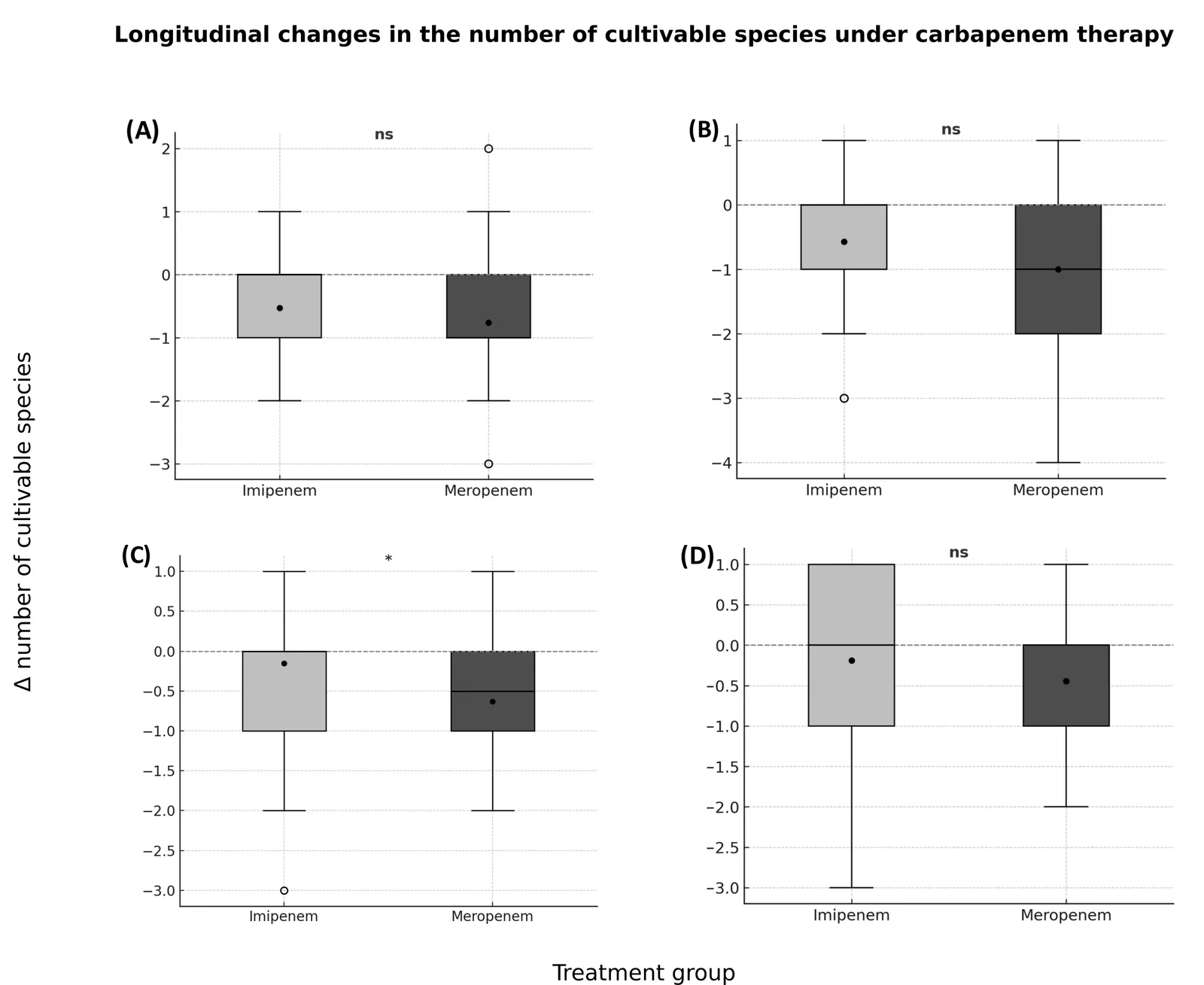
Changes in the number of cultivable species under carbapenem therapy by treatment group. (**A**) Change in the total number of cultivable species recovered on the six culture media between T0 and T1. (**B**) Change in the total number of cultivable species between T0 and T2. (**C**) Change in the number of cultivable *Enterobacterales* species between T0 and T1. (**D**) Change in the number of cultivable *Enterobacterales* species between T0 and T2. Between-group comparisons of paired changes were performed using the Mann–Whitney U test. T0, before the first carbapenem infusion; T1, 48–72 h after treatment initiation; T2, end of therapy.

Between T0 and T1, the number of cultivable *Enterobacterales* species decreased more frequently in the meropenem group than in the imipenem group (57.1% vs 21.9%; *P* = 0.018). This difference was also reflected in paired median changes (Fig. 3C). Changes in the total number of cultivable species across the culture media used in the study followed a similar trend but did not reach statistical significance (Fig. 3A).

Between T0 and T2, reductions in the number of cultivable *Enterobacterales* species and in the total number of cultivable species showed the same directional trend in the meropenem group; however, neither comparison reached statistical significance (Fig. 3B through D).

**Fig 4:**
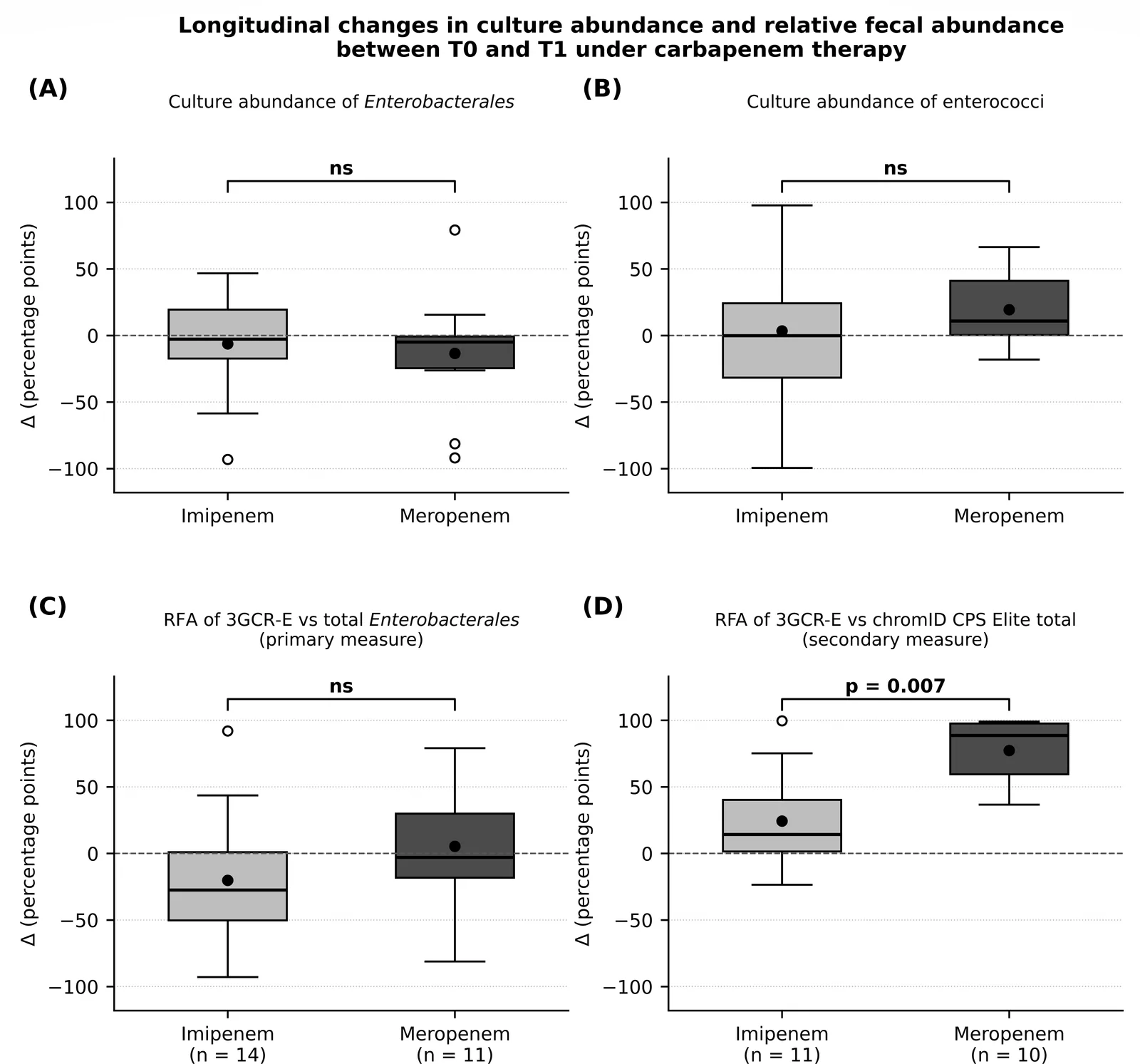
Longitudinal changes in cultivable bacterial populations between T0 and T1, by treatment group. (**A**) Change in the culture abundance of total cultivable Enterobacterales enumerated on Drigalski agar. (**B**) Change in the culture abundance of Enterococci enumerated on chromID CPS Elite agar. (**C**) Change in the relative fecal abundance (RFA) of third-generation cephalosporin-resistant *Enterobacterales* (3GCR-E) calculated relative to total cultivable *Enterobacterales* recovered on Drigalski agar (primary measure). (**D**) Change in the RFA of 3GCR-E calculated relative to aerobic cultivable bacteria recovered on chromID CPS Elite agar (secondary, exploratory measure). Between-group comparisons were performed using the Mann–Whitney U test. T0, before the first carbapenem infusion; T1, 48–72 h after treatment initiation.

Analysis of longitudinal changes in bacterial counts between T0 and T1 showed no significant differences between treatment groups for total Enterobacterales or Enterococci (Fig. 4A and B). Similarly, longitudinal changes in the RFA of 3GCR-E at T1 did not differ significantly between groups when calculated relative to total cultivable *Enterobacterales*, the primary denominator, which remained stable in both groups (Fig. 4C). In a secondary, exploratory analysis using aerobic cultivable bacteria recovered on chromID CPS Elite as denominator, the ratio increased significantly in the meropenem group (Fig. 4D). As the primary ratio remained stable while the secondary ratio increased, this observation corresponds to an increase in the share of *Enterobacterales* within the aerobic cultivable fraction recovered on this medium.

When loss of cultivable species was defined as the disappearance of at least one species between T0 and T1, this occurred in 13 of 52 patients treated with imipenem (25.0%) and 13 of 31 of those treated with meropenem (41.9%) (*P* = 0.143). Likewise, a decrease of at least 2 log₁₀ CFU/mL in *Enterobacterales* counts was observed in 7 of 52 (13.5%) and 9 of 31 (29.0%) patients, respectively (*P* = 0.094). These proportions were calculated among all included patients, patients without an evaluable follow-up sample being counted as not having experienced the event.

### Residual antibiotic detection in rectal samples

UHPLC-HRMS analyses showed marked differences between carbapenems in residual antibiotic detection in rectal samples.

Pharmacological analyses were performed on T1 and T2 samples only, as T0 samples were collected before the first carbapenem infusion. Pharmacological data were missing for 7 of 58 samples (12%) in the imipenem group and 5 of 35 samples (14%) in the meropenem group, leaving 31 of 37 and 22 of 23 evaluable samples at T1, and 20 of 21 and 8 of 12 evaluable samples at T2, in the imipenem and meropenem groups, respectively. Each patient contributed at most one sample per time point.

**Fig 5:**
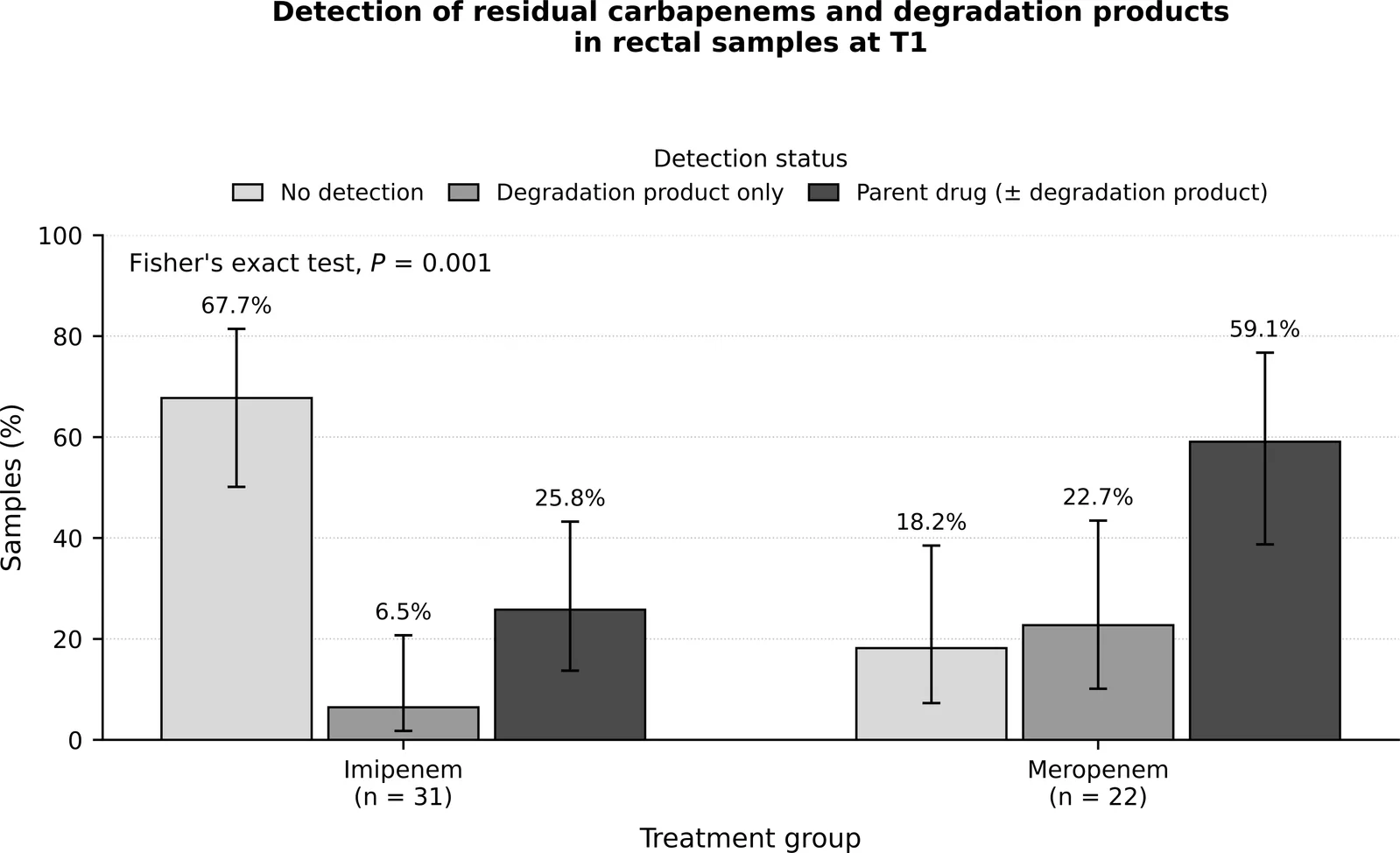
Detection of residual carbapenems and degradation products in rectal samples at T1.

At T1 (48–72 h), meropenem was detected in 13 of 22 evaluable samples (59.1%), with a median concentration of 116.4 µg/L (IQR 19.9–423.5), and its hydrolysis products in 14 of 22 samples (63.6%). By contrast, imipenem was detected in 8 of 31 evaluable samples (25.8%), with a median concentration of 19.7 µg/L (IQR 9.3–100.7), and its hydrolysis products in 6 of 31 samples (19.3%) (*P* = 0.0016) (Fig. 5).

At T2 (end of therapy; median duration, 7 days), meropenem was only rarely detected as the parent compound, whereas its degradation products remained detectable in 7 of 8 evaluable samples (87.5%). Imipenem residues remained uncommon and were detected in two patients, at concentrations of 34.4 and 526.4 µg/L.

### Changes in cultivable bacterial measures according to residual carbapenem detection

**Fig 6:**
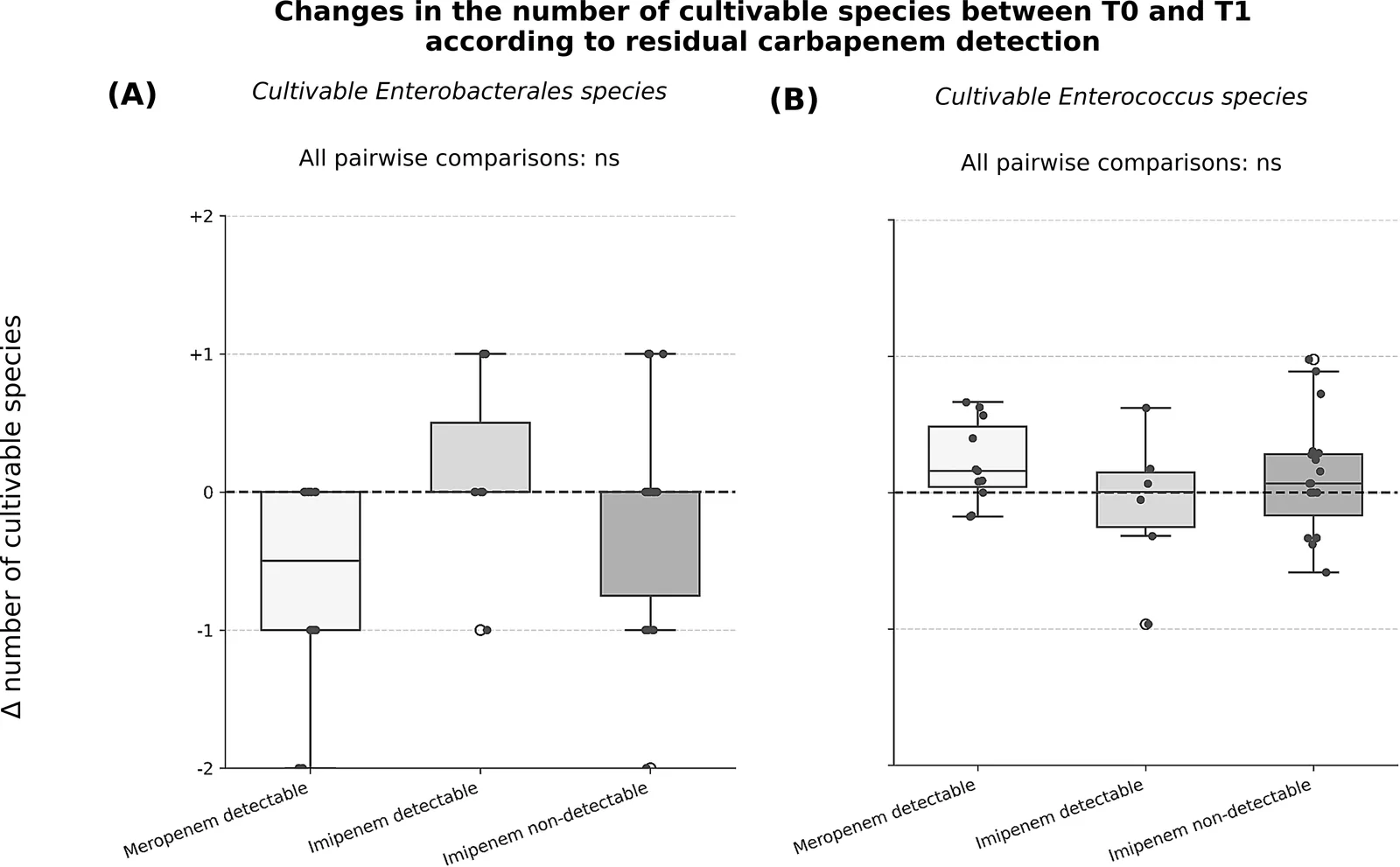
Changes in the number of cultivable *Enterobacterales* species according to fecal antibiotic detection. (**A**) *Enterobacterales*. (**B**) *Enterococcus*.

When analyses were stratified according to residual antibiotic detection, no significant differences in the number of cultivable *Enterobacterales* species were observed between samples without detectable imipenem and those with detectable imipenem or its degradation products. A similar pattern was observed for meropenem (Fig. 6A). By contrast, the number of cultivable *Enterococcus* species tended to increase in samples with detectable meropenem or its degradation products (Fig. 6B).

**Fig 7:**
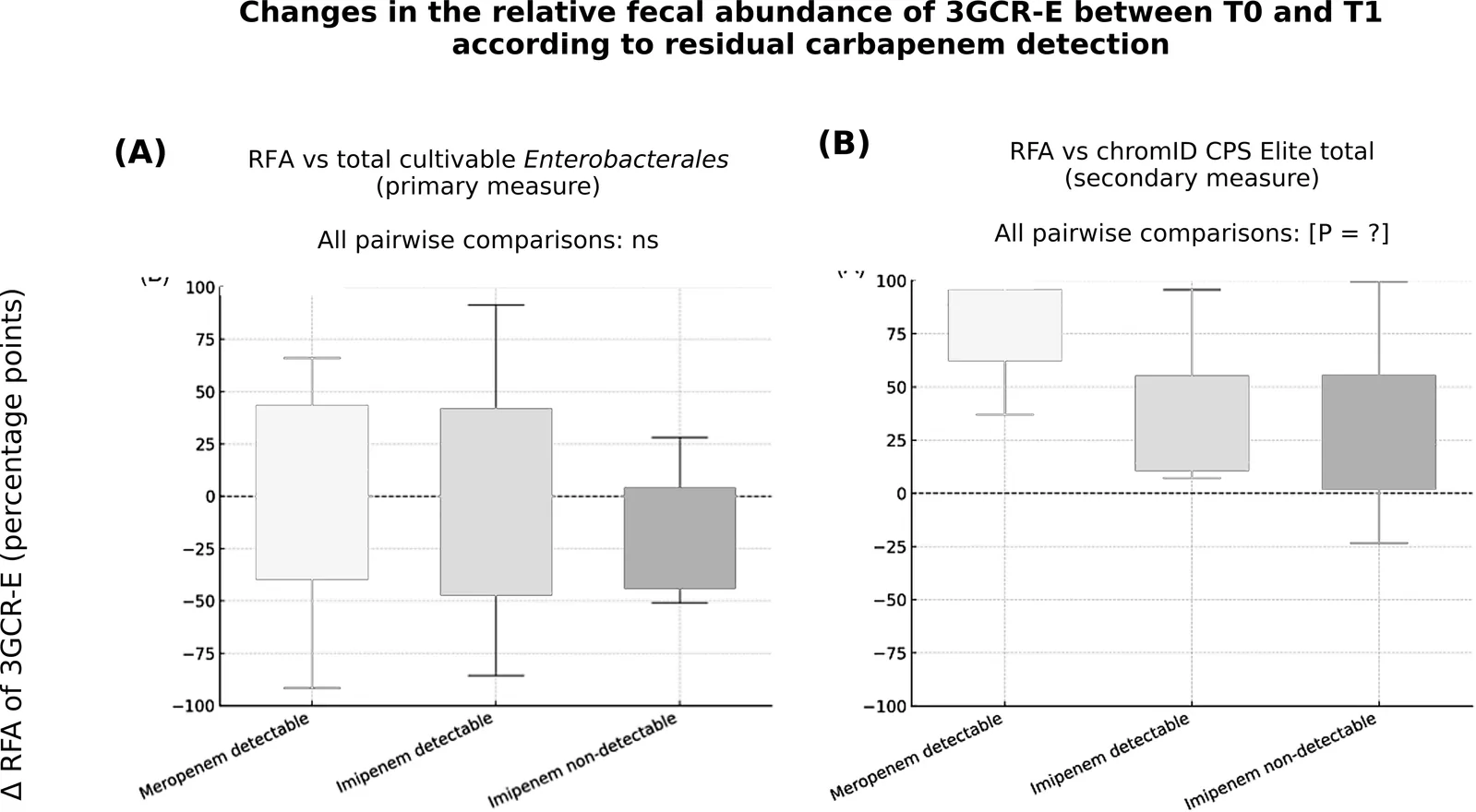
Changes in relative fecal abundance according to treatment group and fecal antibiotic detection. (**A**) RFA versus total cultivable *Enterobacterales*. (**B**) RFA vs chromID CPS Elite total.

Consistent with the longitudinal analyses, changes in the relative fecal abundance of 3GCR-E were further examined according to residual antibiotic detection. The primary ratio, calculated relative to total cultivable *Enterobacterales*, remained stable regardless of antibiotic detection status (Fig. 7A). In the secondary, exploratory analysis using aerobic cultivable bacteria recovered on chromID CPS Elite as denominator, the ratio increased in samples with detectable meropenem or its degradation products (Fig. 7B), again reflecting an increased share of *Enterobacterales* within the aerobic cultivable fraction.

### Emergence of other resistant micro-organisms

Among the 59 patients with paired samples (36 in the imipenem group and 23 in the meropenem group), acquisition was defined as the detection at T1 or T2 of an organism absent from the same patient’s T0 sample. Newly detected 3GCR-E carriage was observed in six patients, including four in the imipenem group and two in the meropenem group (Table 3). Two patients treated with imipenem had newly detected CRE carriage; however, no carbapenemase was identified. A single non-toxigenic *Clostridioides difficile* isolate was recovered from a patient treated with meropenem.

**TABLE 3:** Newly detected carriage of resistant micro-organisms during carbapenem therapy among the 59 patients with paired samples (36 imipenem, 23 meropenem)^*a*^^,^^*b*^

Emergent organism	Total (*n* = 59)	Imipenem (*n* = 36)	Meropenem (*n* = 23)	*P-*value
3GCR-E, *n* (%)	6 (10.2)	4 (11.1)	2 (8.7)	1.000
CRE, *n* (%)	2 (3.4)	2 (5.5)	0	0.516
VRE, *n* (%)	0 (0)	0	0	NA
*Clostridioides difficile*, *n* (%)	1 (1.7)	0	1 (4.3)	1.000
Carbapenem-resistant *P. aeruginosa, n* (%)	0	0	0	NA
Any MDR organism, *n* (%)	9 (15.3)	6 (16.7)	3 (13.0)	0.739

^*a*^ Acquisition was defined as the detection at T1 (48–72 h after treatment initiation) or T2 (end of therapy) of an organism not recovered from the same patient’s baseline (T0) sample. 3GCR-E, third-generation cephalosporin-resistant *Enterobacterales*; CRE, carbapenem-resistant *Enterobacterales*; VRE, vancomycin-resistant enterococci.

^*b*^ Not applicable (NA), *P* value not calculated because no events occurred in either group.

## DISCUSSION

### Comparative intestinal microbiological changes under imipenem and meropenem exposure

This study showed that imipenem and meropenem were associated with different patterns of residual intestinal exposure and changes in targeted cultivable intestinal bacterial populations in hospitalized patients. Compared with meropenem, imipenem was associated with lower residual antibiotic detection in rectal samples and with more limited changes in the cultivable intestinal bacterial populations assessed in this study, in line with previous culture-based and metagenomic observations suggesting a lower intestinal ecological impact of imipenem (11, 30, 31).

In contrast, meropenem exposure was associated with a higher relative fecal abundance (RFA) of third-generation cephalosporin-resistant Enterobacterales (3GCR-E) and a more frequent decrease in the number of cultivable *Enterobacterales* species. Taken together, these findings support the existence of differential microbiological effects between carbapenems on the targeted cultivable intestinal bacterial populations assessed here and suggest potential relevance for antimicrobial stewardship in high-risk patients.

### Role of pharmacokinetics and dosing

The differential effects observed in this study are likely related, at least in part, to differences in the pharmacokinetic profiles of imipenem and meropenem. Imipenem is predominantly eliminated by renal clearance, with minimal biliary excretion, whereas meropenem undergoes greater biliary elimination and intestinal exposure (14, 15, 17, 18). In our cohort, meropenem was administered at a higher median daily dose than imipenem (3 g/day [IQR 3–4] vs 2 g/day [IQR 2–2], *P* < 0.001). These correspond to the standard regimens of 1 g every 8 h and 500 mg every 6 h, respectively, and the difference, therefore, reflects the reference posologies of the two agents rather than an imbalance in prescribing between groups. Nevertheless, because higher daily doses may increase intraluminal exposure independently of biliary excretion, dose and pharmacokinetic profile cannot be disentangled in an observational design, and the contribution of each to the more frequent detection of meropenem residues cannot be quantified here. Altogether, these observations are consistent with the concept that biliary excretion and intraluminal antibiotic exposure contribute substantially to the intestinal ecological effects of β-lactams (14, 15, 17, 18).

These observations raise the question of how other carbapenems would behave. Ertapenem is instructive in this respect because its spectrum and its intestinal exposure point in opposite directions. Despite a narrower spectrum than meropenem, ertapenem reaches substantial fecal concentrations, with about 10% of an administered dose recovered in feces, and in healthy volunteers, this was accompanied by marked reductions in *Escherichia coli*, *Bacteroides* spp., and bifidobacteria and by overgrowth of enterococci and yeasts—an intestinal impact comparable to that of ceftriaxone rather than to that of the other carbapenems (35). If intraluminal exposure rather than spectrum drives the ecological effect, as our data suggest, ertapenem would be expected to produce changes at least as marked as meropenem in the populations examined here, and possibly greater enterococcal expansion given its negligible activity against *Enterococcus* spp.

The consequences for non-fermenting Gram-negative bacilli are less predictable. Ertapenem has no clinically relevant activity against *Pseudomonas aeruginosa* or *Acinetobacter* spp., so any increase in these populations would reflect release from competition rather than direct selection. Ertapenem can select OprD-deficient *P. aeruginosa* mutants *in vitro*, although this selectivity is thought to be limited under clinical conditions (36), and hospital-level studies of ertapenem use and antipseudomonal carbapenem susceptibility have yielded conflicting results (37). Our approach, which combines targeted culture with direct measurement of intraluminal drug exposure in the same sample, would be well suited to testing these hypotheses prospectively.

### Changes in enterococci and in targeted cultivable bacterial populations

Enterococci are intrinsically less susceptible than many other intestinal bacteria to broad-spectrum β-lactams and may expand under antibiotic pressure when competing populations are reduced (28). This susceptibility differs between the two agents studied: imipenem has stronger intrinsic activity against *Enterococcus faecalis* than meropenem (16, 19–21). Any difference in enterococcal populations between the two treatment groups is, therefore, an anticipated consequence of this spectrum difference rather than a novel observation. Consistent with this, enterococcal differences in our cohort were modest overall: neither culture abundance between treatment groups (Fig. 4B) nor the number of cultivable *Enterococcus* species according to residual carbapenem detection (Fig. 6B) differed significantly, and the only notable species-level distinction, aminopenicillin-resistant *E. faecium* at T1 (Table 2), aligned with imipenem’s known anti-enterococcal advantage. Interpretation is further limited by concomitant exposure to agents active against Gram-positive organisms in a subset of patients. We, therefore, do not present these findings as a principal result. Rather, our conclusions rest on the comparison that is not explained by intrinsic spectrum differences, namely, the *Enterobacterales* and the residual intraluminal drug exposure measured in the same samples.

### Persistence of 3GCR-*Enterobacterales* carriage

Despite these changes, carriage of 3GCR-E persisted under both carbapenems during treatment. No clear reduction in carriage was observed with either imipenem or meropenem, in keeping with previous observations that carbapenem exposure does not reliably eradicate intestinal carriage of resistant Enterobacterales (25). Persistence of carriage is likely multifactorial and may depend on host-related factors, previous antimicrobial exposure, bacterial fitness, and ecological resilience. Importantly, our data suggest that measurable changes in cultivable intestinal bacterial populations may occur without an apparent benefit in terms of reducing the intestinal reservoir of resistant Enterobacterales.

### Integration of pharmacological and microbiological data

A major strength of this study is the integration of microbiological outcomes with UHPLC-HRMS detection of residual carbapenem exposure in rectal samples. This approach strengthens the biological plausibility of our findings by linking detectable antibiotic residues with changes in cultivable bacterial measures. The gradient observed across exposure groups, from limited changes in patients without detectable imipenem residues to more marked changes in patients with detectable meropenem residues, supports an exposure-dependent association with changes in cultivable bacterial measures. This exposure-dependent gradient was observed for the secondary ratio, calculated relative to aerobic cultivable bacteria recovered on chromID CPS Elite, and should, therefore, be regarded as exploratory; the primary ratio, calculated relative to total cultivable *Enterobacterales*, remained stable across exposure groups. This pharmacological dimension is often missing from studies evaluating the intestinal effects of antibiotics.

One methodological point deserves attention. Imipenem degrades faster than meropenem under the storage conditions used, so differential analytical stability could in principle contribute to the lower detection of the parent compound in the imipenem group. Two observations argue against this being the main explanation. First, hydrolysis products remain detectable after the parent compound has degraded, and the composite measure of parent drug or hydrolysis product, which is largely insensitive to this bias, still differed markedly between groups, being positive in 10 of 31 evaluable samples in the imipenem group and 18 of 22 in the meropenem group at T1 (Fig. 5). Second, the direction of the difference is that predicted by the respective biliary elimination of the two agents.

The direction of this finding is expected: the greater biliary elimination and fecal excretion of meropenem make higher intraluminal concentrations predictable, and our data confirm it. More specifically, the pharmacological data themselves are informative independently of the microbiological measures: meropenem was detected in a minority of patients rather than in nearly all of them, and its degradation products remained detectable at the end of therapy in most samples. Such interindividual variability in intraluminal exposure is not predicted by average pharmacokinetic parameters and allows microbiological findings to be stratified on measured rather than assumed intestinal exposure.

### Place of culture-based approaches in ecological studies of antimicrobial resistance

Our study relied on targeted culture-based methods rather than metagenomic profiling. As such, it does not allow characterization of the global gut microbiome, particularly strict anaerobes and uncultured taxa, and our results should, therefore, not be interpreted as reflecting overall microbiome disruption. However, culture-based approaches remain highly informative for ecological studies focused on antimicrobial resistance because they provide access to viable isolates, phenotypic resistance profiles, and quantitative data on clinically relevant subdominant populations such as resistant Enterobacterales and enterococci.

More broadly, current literature indicates that culture-based approaches and metagenomics should be viewed as complementary rather than competing strategies. Culturomics has substantially expanded the repertoire of cultured gut bacteria and can recover taxa not readily captured by sequencing alone, while metagenomics provides a broader view of community composition and functional potential (32, 38). Recent work further emphasizes the synergy between both approaches, particularly when the objective is to connect compositional signals to experimentally tractable isolates for functional validation (32). In that perspective, our targeted culture-based strategy was appropriate for addressing clinically relevant endpoints related to intestinal carriage, resistant Enterobacterales burden, and residual antibiotic exposure although it does not replace metagenomic investigation of global microbiome changes (32, 33, 38).

### Additional mechanistic considerations

These variations in intestinal antibiotic-associated changes may also reflect local antibiotic concentrations below the threshold required to exert measurable selective effects, consistent with concentration-dependent phenomena previously described for fluoroquinolones (39). To mitigate such intestinal microbiological effects, adjunctive strategies such as oral β-lactamases (e.g., ribaxamase) could be considered, as they have been shown to protect the gut microbiota from exposure to intravenous β-lactams and to delay the emergence of antimicrobial resistance (40). Alternatively, partial degradation of carbapenems by gut microbiota members capable of producing carbapenemases, such as certain *Bacteroides* species, may also contribute to the observed interindividual variability in intestinal exposure (41, 42).

### Clinical implications and perspectives

Our data locate the difference between the two agents at a specific point in the treatment course. The largest between-group differences, both in the RFA of 3GCR-E and in the number of cultivable *Enterobacterales* species, were observed at T1, 48–72 h after initiation, whereas no significant difference was detected at T2. T1, therefore, appears to be the interval during which the intestinal reservoir is most perturbed, and plausibly the interval during which a patient is most vulnerable to endogenous infection and most likely to shed resistant organisms into the hospital environment. This has a practical implication: interventions intended to preserve colonization resistance, and infection-control measures intended to limit cross-transmission, would be expected to matter most during the first days of carbapenem therapy rather than at its end.

Two caveats apply. First, our culture panel targeted a defined set of organisms, and other populations known to expand under carbapenem pressure were not sought, including *Candida* spp., *Stenotrophomonas maltophilia*, non-fermenting Gram-negative bacilli other than *P. aeruginosa*, and carbapenemase-producing anaerobes. The changes described here are, therefore, likely to underestimate the range of organisms that expand during treatment. Second, the absence of a significant difference at T2 should not be interpreted as stabilization: only 21 and 12 patients contributed evaluable samples at that time point, the RFA of 3GCR-E was 40% vs 12%, and the study was not powered to exclude a difference of that magnitude.

From an antimicrobial stewardship perspective, these findings suggest that when both agents are clinically appropriate, imipenem may be associated with less pronounced intestinal microbiological changes in the measures assessed here than meropenem. This observation may be particularly relevant in critically ill patients, in whom preservation of colonization resistance and limitation of resistant bacterial expansion are important objectives. These considerations support antimicrobial stewardship strategies that take intestinal consequences into account, and specifically during the early treatment interval around T1, when the reservoir of resistant *Enterobacterales* is most enriched and the risk of endogenous infection and onward transmission is likely to be highest. Our results also support further evaluation of quantitative intestinal markers, such as the relative fecal abundance of 3GCR-E, as indicators of subsequent infection or transmission risk, as suggested in recent ICU and urinary tract infection studies (43, 44).

### Limitations

This study has several limitations. First, it was based on rectal swabs rather than stool samples. Although rectal swabs may not fully reflect overall fecal microbial composition, they provided a pragmatic and standardized approach for serial sampling in hospitalized patients, particularly in intensive care settings where sufficient bowel transit is often lacking and stool collection is frequently not feasible. Second, our analyses relied on targeted culture-based methods and, therefore, did not capture the global gut microbiome and uncultured organisms. In particular, the absence of metagenomic analysis precludes any conclusion regarding whole-community composition, functional pathways, or overall microbiome disruption (32, 33, 38). In addition, the absence of a control group not exposed to carbapenems precludes distinguishing carbapenem-related changes from temporal changes related to hospitalization, critical illness, or other concurrent exposures. Third, our culture panel was designed for the detection and enumeration of specific organism groups rather than for community-level ecological assessment. Although chromID CPS Elite contains no antimicrobial supplement and supports the growth of a broad range of aerobic organisms including Gram-positive cocci, its performance has been validated for urinary rather than fecal specimens. No non-selective medium and no anaerobic culture other than for *C. difficile* were included, so strict anaerobes were not recoverable by design. The use of enrichment and selective media may additionally have influenced the recovery and quantification of some bacterial populations. Accordingly, the number of species recovered should be interpreted as culture recovery on a defined panel and not as a measure of intestinal bacterial diversity. Fourth, the two carbapenems were administered at their respective standard daily doses, which differ; consequently, dose and intrinsic pharmacokinetic profile were confounded by design, and our data cannot establish the relative contribution of each to the differences in residual intestinal exposure observed. Fifth, although a substantial proportion of patients received concomitant antibiotic therapy, this consisted almost exclusively of parenteral aminoglycosides, with intravenous vancomycin in five patients distributed evenly between groups; agents with a major impact on intestinal *Enterobacterales* or anaerobes, such as fluoroquinolones and metronidazole, were not administered. Residual confounding by concomitant antibiotic exposure is, therefore, unlikely although it cannot be formally excluded. Sixth, pharmacological analyses were not available for all samples because of missing or non-exploitable specimens. Seventh, approximately 10% of samples were stored at −80°C before analysis, which may have affected bacterial recovery. Finally, because the study population consisted predominantly of critically ill patients, the generalizability of these findings to other hospitalized or non-hospitalized populations remains uncertain.

These limitations should be balanced against several strengths, including the prospective design, the direct comparison between imipenem and meropenem, the repeated within-patient sampling, and the integration of microbiological and pharmacological data.

### Conclusion

In this prospective comparative study, imipenem and meropenem showed differential effects on the targeted cultivable intestinal bacterial populations and on residual antibiotic detection in rectal samples. Compared with meropenem, imipenem was associated with lower residual intestinal exposure and more limited changes in cultivable bacterial measures. These differences were concentrated at 48–72 h after treatment initiation, identifying the early treatment interval as the period during which the intestinal reservoir of resistant *Enterobacterales* is most enriched and, plausibly, during which the risk of endogenous infection and onward transmission is highest. These findings suggest that pharmacokinetic differences between carbapenems contribute to their differential intestinal effects and support antimicrobial stewardship and infection-control strategies that take this early window into account when both agents are clinically appropriate.

## Data Availability

The data that support the findings of this study are available from the corresponding author upon reasonable request.

## SUPPLEMENTAL MATERIAL

Figure S1 spectrum.01235-26-s0001.tiff Study protocol.

Table S1 spectrum.01235-26-s0002.docx Identification of *Enterobacterales* species and associated third-generation cephalosporin (3GC) resistance mechanisms (ESBL, AmpC) at each sampling time point (T0, T1, T2) for each individual patient, separately for the imipenem group (Table S1, *n* = 52) and the meropenem group (Table S2, *n* = 31).

ASM does not own the copyrights to Supplemental Material that may be linked to, or accessed through, an article. The authors have granted ASM a non-exclusive, world-wide license to publish the Supplemental Material files. Please contact the corresponding author directly for reuse.
