# Supplementary material for: Comparative impact of imipenem and meropenem on intestinal carriage and relative fecal abundance of multidrug-resistant bacteria, with detection of residual antibiotics in rectal swabs
Source: Microbiol Spectr. 2026 Sep 17;14(10):e01235-26. doi: 10.1128/spectrum.01235-26 (PMC13689392; doi:10.1128/spectrum.01235-26)
Supplement: Supplementary file 1 [file spectrum.01235-26-s0002.docx]

**Supplemental File**

**Table S1**. Identification of *Enterobacterales* in each patient in the imipenem group at different times and associated 3GC resistance mechanisms

| **Patient** | **Time** | ***Enterobacterales***  **diversity** | **Identification of *Enterobacterales*** | **3GCR** | **Resistance mechanism** |
| --- | --- | --- | --- | --- | --- |
| **1** | T0  T1  T2 | 2  2  NA | *E. coli, E. cloacae*  *E. coli, E. cloacae* | Yes  Yes | ESBL  ESBL |
| **2** | T0  T1  T2 | 3  NA  NA | *C. freundii, E. cloacae, E. coli* | Yes | ESBL |
| **3** | T0  T1  T2 | 1  1  1 | *K. pneumoniae*  *K. pneumoniae* | No  No |  |
| **4** | T0  T1  T2 | 0  1  1 | *E. coli*  *E. coli* | No  No |  |
| **5** | T0  T1  T2 | 2  NA  NA | *E. cloacae, h.alvei* | Yes | AmpC |
| **6** | T0  T1  T2 | 2  NA  NA | *K. aerogenes, E. coli* | Yes | AmpC |
| **7** | T0  T1  T2 | 2  2  3 | *K. pneumoniae, E. coli*  *K. pneumoniae, E. coli*  *K. pneumoniae, m. morganii* | Yes  Yes  Yes | ESBL  ESBL  ESBL |
| **8** | T0  T1  T2 | 3  NA  NA | *E. coli, K. pneumoniae, k.aerogenes* | Yes | ESBL |
| **9** | T0  T1  T2 | 2  NA  NA | *K. pneumoniae, E. coli* | Yes | ESBL |
| **10** | T0  T1  T2 | 2  2  2 | *K. pneumoniae, E. coli*  *K. pneumoniae, E. coli*  *K. pneumoniae, E. coli* | Yes  Yes  Yes | ESBL  ESBL  ESBL |
| **11** | T0  T1  T2 | NA  1  NA | *E. coli* | Yes | ESBL |
| **12** | T0  T1  T2 | 3  NA  2 | *E. cloacae, K. pneumoniae, E. coli*  *H. alvei, E. coli* | Yes  No | ESBL |
| **13** | T0  T1  T2 | 1  NA  1 | *E. coli*  *E. coli* | No  No |  |
| **14** | T0  T1  T2 | 1  1  NA | *k. aero*  *E. coli* | No  Yes | ESBL |
| **15** | T0  T1  T2 | NA  1  NA | *E. coli* | Yes | ESBL |
| **16** | T0  T1  T2 | 2  1  1 | *E. coli, K. pneumoniae*  *k. oxycota*  *k. oxycota* | No  No  No |  |
| **17** | T0  T1  T2 | 2  0  0 | *E. coli., k. oxycota* | No |  |
| **18** | T0  T1  T2 | 3  0  0 | *E. coli, E. cloacae, K. pneumoniae* | Yes | ESBL |
| **19** | T0  T1  T2 | 0  1  1 | *P. mirabilis*  *P. mirabilis* | No  No |  |
| **20** | T0  T1  T2 | 0  1  1 | *P. mirabilis*  *P. mirabilis* | No  No |  |
| **21** | T0  T1  T2 | NA  1  NA | *E. coli* | No |  |
| **22** | T0  T1  T2 | 4  4  4 | *E. cloacae, P. vulgaris, E. coli, K. pneumoniae*  *M.morganii, P. vulgaris, E. cloacae, E. coli*  *E. cloacae, P. vulgaris, K. pneumoniae, E. coli* | Yes  Yes  Yes | ESBL  AmpC  AmpC |
| **23** | T0  T1  T2 | 2  NA  1 | *E. coli, K. pneumoniae*  *E. coli* | Yes  No | ESBL |
| **24** | T0  T1  T2 | 1  2  2 | *E. cloacae*  *E. cloacae, p. mirabilis*  *E. cloacae, p. mirabilis* | Yes  Yes  Yes | ESBL  ESBL  ESBL |
| **25** | T0  T1  T2 | 1  0  0 | *E. coli* | Yes | ESBL |
| **26** | T0  T1  T2 | 0  0  NA |  |  |  |
| **27** | T0  T1  T2 | 1  0  0 | *C. freundii* | Yes | AmpC |
| **28** | T0  T1  T2 | 2  2  2 | *E. cloacae, E. coli*  *E. cloacae, E. coli*  *E. cloacae, E. coli* | Yes  Yes  Yes | ESBL  ESBL  ESBL |
| **29** | T0  T1  T2 | 2  3  NA | *E. coli, K. pneumoniae*  *E. coli, k. p., E. cloacae* | No  Yes | ESBL |
| **30** | T0  T1  T2 | 1  0  NA | *E. coli* | No |  |
| **31** | T0  T1  T2 | 1  NA  NA | *k. aero* | Yes | ESBL |
| **32** | T0  T1  T2 | 3  NA  NA | *E. coli, H. alvei, E. cloacae* | Yes | ESBL |
| **33** | T0  T1  T2 | 1  1  NA | *E. cloacae*  *E. cloacae* | Yes  Yes | ESBL  ESBL |
| **34** | T0  T1  T2 | 1  0  NA | *E. coli* | Yes | ESBL |
| **35** | T0  T1  T2 | 1  1  1 | *E. coli*  *E. coli*  *E. coli* | No  No  No |  |
| **36** | T0  T1  T2 | 1  1  NA | *E. coli*  *E. coli* | Yes  Yes | ESBL  ESBL |
| **37** | T0  T1  T2 | 1  1  NA | *K. pneumoniae*  *K. pneumoniae* | Yes  Yes | ESBL  ESBL |
| **38** | T0  T1  T2 | 0  NA  1 | *E. cloacae* | Yes | ESBL |
| **39** | T0  T1  T2 | 0  0  NA |  |  |  |
| **40** | T0  T1  T2 | 0  0  NA |  |  |  |
| **41** | T0  T1  T2 | 1  1  1 | *K. pneumoniae*  *K. pneumoniae*  *K. pneumoniae* | Yes  Yes  Yes | ESBL  ESBL  ESBL |
| **42** | T0  T1  T2 | 0  0  0 |  |  |  |
| **43** | T0  T1  T2 | 3  3  NA | *K. oxycocta, P. mirabilis, E. cloacae*  *K. pneumoniae, P. mirabilis, e. clocae* | Yes  Yes | ESBL  ESBL |
| **44** | T0  T1  T2 | 0  1  NA | *E. coli* | Yes | ESBL |
| **45** | T0  T1  T2 | 2  NA  NA | *E. cloacae, E. coli* | Yes | AmpC |
| **46** | T0  T1  T2 | 0  0  NA |  |  |  |
| **47** | T0  T1  T2 | 1  NA  NA | *E. coli* | No |  |
| **48** | T0  T1  T2 | NA  2  NA | *K. aerogenes, E. coli* | Yes | AmpC |
| **49** | T0  T1  T2 | 1  1  NA | *E. coli*  *E. coli* | Yes  Yes | ESBL  ESBL |
| **50** | T0  T1  T2 | 0  NA  NA |  |  |  |
| **51** | T0  T1  T2 | NA  0  NA |  |  |  |
| **52** | T0  T1  T2 | 1  NA  NA | *E. coli* | Yes | ESBL |

**Table S2**. Identification of *Enterobacterales* in each patient in the meropenem group at different times and associated 3GC resistance mechanisms

| **Patient** | **Time** | ***Enterobacterales***  **diversity** | **Identification of *Enterobacterales*** | **3GC R** | **Resistance mechanism** |
| --- | --- | --- | --- | --- | --- |
| **1** | T0  T1  T2 | 1  1  1 | *E. coli*  *E. coli*  *E. coli* | Yes  Yes  Yes | ESBL  ESBL  ESBL |
| **2** | T0  T1  T2 | 1  0  2 | *E. coli*  *K. aerogenes* | No  Yes | AmpC |
| **3** | T0  T1  T2 | 1  NA  NA | *E. coli* | No |  |
| **4** | T0  T1  T2 | NA  NA  1 | *C. braakii* | Yes | AmpC |
| **5** | T0  T1  T2 | 3  2  2 | *E. coli, E. cloacae, K. pneumoniae*  *E. coli, E. cloacae*  *E. coli, E. cloacae* | Yes  Yes  Yes | ESBL  ESBL  ESBL |
| **6** | T0  T1  T2 | 2  NA  2 | *E. coli, K. pneumoniae*  *E. coli, E. cloacae* | Yes  Yes | ESBL  ESBL |
| **7** | T0  T1  T2 | 2  1  0 | *E. coli, K. pneumoniae*  *K. aerogenes* | No  Yes | ESBL |
| **8** | T0  T1  T2 | 2  0  0 | *E. coli, K. pneumoniae* | No |  |
| **9** | T0  T1  T2 | NA  0  0 |  |  |  |
| **10** | T0  T1  T2 | 1  1  NA | *E. coli*  *E. coli* | No |  |
| **11** | T0  T1  T2 | 2  0  NA | *E. coli, P. vulgaris* | No |  |
| **12** | T0  T1  T2 | 0  NA  0 |  |  |  |
| **13** | T0  T1  T2 | 1  0  NA | *E. cloacae* | Yes | ESBL |
| **14** | T0  T1  T2 | 3  3  NA | *E. coli, E. cloacae, P. mirabilis*  *E. coli, E. cloacae, P. mirabilis* | Yes  Yes | ESBL  ESBL |
| **15** | T0  T1  T2 | 2  1  NA | *K. pneumoniae, E. coli*  *E. coli* | Yes  Yes | ESBL  ESBL |
| **16** | T0  T1  T2 | 2  0  NA | *E. coli, K. pneumoniae* | No |  |
| **17** | T0  T1  T2 | 2  0  NA | *E. coli., M. morganii* | No |  |
| **18** | T0  T1  T2 | 2  2  NA | *E. coli, K. pneumoniae*  *E. coli, K. pneumoniae* | Yes  Yes | ESBL  ESBL |
| **19** | T0  T1  T2 | 1  1  1 | *E. cloacae*  *E. cloacae*  *E. cloacae* | Yes  Yes  Yes | ESBL  ESBL  ESBL |
| **20** | T0  T1  T2 | 2  1  NA | *E. coli, K. pneumoniae*  *E. coli* | Yes  Yes | ESBL  ESBL |
| **21** | T0  T1  T2 | 0  NA  NA |  |  |  |
| **22** | T0  T1  T2 | NA  0  0 |  |  |  |
| **23** | T0  T1  T2 | 1  1  NA | *K. pneumoniae*  *K. pneumoniae* | Yes  Yes | ESBL  ESBL |
| **24** | T0  T1  T2 | 2  1  NA | *E. cloacae, E. coli*  *E. cloacae* | Yes  Yes | AmpC  AmpC |
| **25** | T0  T1  T2 | 1  1  NA | *E. cloacae*  *E. cloacae* | Yes  Yes | AmpC |
| **26** | T0  T1  T2 | 1  2  1 | *M. morganii*  *M. morganii, E. coli*  *M. morganii* | No  No  Yes | ESBL |
| **27** | T0  T1  T2 | 2  NA  NA | *E. coli, E. cloacae* | Yes | AmpC |
| **28** | T0  T1  T2 | 1  NA  NA | *E. coli* | No |  |
| **29** | T0  T1  T2 | 1  0  NA | *E. coli* | Yes | ESBL |
| **30** | T0  T1  T2 | 1  1  NA | *K. pneumoniae*  *E. coli* | Yes  Yes | ESBL  ESBL |
| **31** | T0  T1  T2 | 2  NA  NA | *E. coli, H. alvei* | Yes | ESBL |
